# Inhibiting endoplasmic reticulum stress alleviates perioperative neurocognitive disorders by reducing neuroinflammation mediated by NLRP3 inflammasome activation

**DOI:** 10.1111/cns.70049

**Published:** 2024-10-21

**Authors:** Fanbing Meng, Jian Song, Xinwei Huang, Meixian Zhang, Xiaoxiao Sun, Qi Jing, Silu Cao, Zheng Xie, Qiong Liu, Hui Zhang, Cheng Li

**Affiliations:** ^1^ Department of Anesthesiology and Perioperative Medicine, Shanghai Key Laboratory of Anesthesiology and Brain Functional Modulation, Clinical Research Center for Anesthesiology and Perioperative Medicine, Translational Research Institute of Brain and Brain‐Like Intelligence Shanghai Fourth People's Hospital, School of Medicine, Tongji University Shanghai China

**Keywords:** anesthesia and surgery, endoplasmic reticulum stress, neuroinflammation, NLRP3 inflammasome, perioperative neurocognitive disorders

## Abstract

**Aim:**

The aim of this study is to explore the key mechanisms of perioperative neurocognitive dysfunction (PND) after anesthesia/surgery (A/S) by screening hub genes.

**Methods:**

Transcriptome sequencing was conducted on hippocampal samples obtained from 18‐month‐old C57BL/6 mice assigned to control (Ctrl) and A/S groups. The functionality of differentially expressed genes (DEGs) was investigated using Metascape. Hub genes associated with changes between the two groups were screened by combining weighted gene coexpression network analysis within CytoHubba. Reverse transcription PCR and western blotting were used to validate changes in mRNA and protein expression, respectively. NLRP3 inflammasome activation was detected by western blotting and ELISA. Tauroursodeoxycholic acid (TUDCA), an inhibitor of endoplasmic reticulum (ER) stress, was administrated preoperatively to explore its effects on the occurrence of PND. Immunofluorescence analysis was performed to evaluate the activation of astrocytes and microglia in the hippocampus, and hippocampus‐dependent learning and memory were assessed using behavioral experiments.

**Results:**

In total, 521 DEGs were detected between the control and A/S groups. These DEGs were significantly enriched in biological processes related to metabolic processes and their regulation. Four hub genes (*Hspa5*, *Igf1r*, *Sfpq*, and *Xbp1*) were identified. Animal experiments have shown that mice in the A/S group exhibited cognitive impairments accompanied by increased *Hspa5* and *Xbp1* expression, ER stress, and activation of NLRP3 inflammasome.

**Conclusions:**

Inhibiting ER stress alleviated cognitive impairment in A/S mice; particularly, ER stress induced by A/S results in NLRP3 inflammasome activation and neuroinflammation. Moreover, the preoperative administration of TUDCA inhibited ER stress, NLRP3 inflammasome activation, and neuroinflammation.

## Graphical abstract

Anesthesia and surgery‐induced upregulation of *Hspa5* and *Xbp1* gene expression subsequently led to ER stress, NLRP3 inflammasome activation, and neuroinflammation, potentially contributing to cognitive deficits in aged mice.

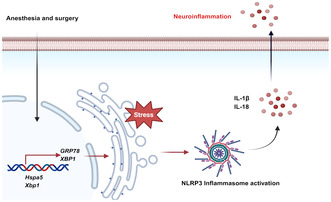

## 1 INTRODUCTION

The number of older patients (age >60 years) undergoing anesthesia and surgery (A/S) has significantly increased with the aging of the global population.^1^, ^2^, ^3^ Perioperative neurocognitive disorders (PND), the most prevalent neurological complication after A/S, is characterized by noticeable memory and cognitive decline, particularly in older patients.^2^, ^4^ The incidence of PND in older patients with hip fractures ranges from 35% to 65%. PND lead to an increase in medical expenditure and prolonged hospital stay, while elevating the risk of developing Alzheimer's disease (AD) and other forms of dementia.^5^ Yet, there is currently no established or effective pharmacological treatment for PND, and the underlying molecular mechanisms remain unclear.

Recent evidence has shown that the microglia and astrocytes that mediate neuroinflammation after surgery are crucial factors for the development of PND.^6^, ^7^ Nucleotide‐binding oligomerization domain (NOD)‐like receptors (NLRs), including NODs, play a crucial role in innate immunity.^8^ One significant component of the NLR family is the NLRP3 inflammasome, comprising the NLRP3 protein, apoptosis‐associated speck‐like protein (ASC), and pro‐caspase‐1. This complex regulates the maturation and release of inflammatory factors, interleukin (IL)‐1β and IL‐18, through the activation of caspase‐1.^8^, ^9^ Hyperactivation of the NLRP3 inflammasome is implicated in a spectrum of inflammation‐related diseases.^9^, ^10^, ^11^ Moreover, recent studies have revealed an association between NLRP3 inflammasome activation and a decline in learning and memory that could be reversed by inhibiting the former.^12^

The accumulation of misfolded proteins in the endoplasmic reticulum (ER) of neurons and neuroglia is a common characteristic of numerous neurodegenerative diseases.^13^ In response to ER stress, cells initiate a highly conserved cellular stress response known as the unfolded protein response (UPR) to restore homeostasis^14^; it is crucial for preserving important biological processes in the brain during cellular stress.^14^ Moderate ER stress enhances cellular protection and increases ER adaptability, inducing a hormetic response.^15^ However, extended ER stress in neurodegenerative disorders compromises the protective mechanisms of the UPR, thereby activating inflammatory and apoptotic pathways that contribute to nervous system toxicity.^15^ ER stress is commonly observed in various experimental models of AD and is strongly associated with memory and cognitive impairment.^16^ Accumulating evidence suggests that ER stress and NLRP3 inflammasome activation form the pathological basis of various inflammatory diseases.^17^, ^18^ Although many NLRP3 regulations and networks were studied, the relationship between NLRP3 inflammasome activation and ER stress in PND was still poorly understood. Here, we aimed to explore the key mechanisms of neurocognitive dysfunction after A/S by screening hub genes in the hippocampus of aged mice with PND.

## 2 METHODS

### 2.1 Animals and experimental design

Eighteen‐month‐old male mice weighing 26–36 g were used in this study. The mice were housed in cages and maintained in standard housing conditions (23 ± 1°C, 50% humidity, 12 h light–dark cycle) with food and water for 14 days. All behavioral tests were conducted during the daytime to ensure consistency in the assessment conditions. In the first experiment, aged mice were randomly assigned to control (Ctrl) group (not being exposed to laparotomy) and laparotomy (A/S) group (being subjected to laparotomy). The hippocampus were harvested for RNA‐sequencing (RNA‐seq) (*n* = 12), quantitative polymerase chain reaction (qPCR) (*n* = 6), western blotting (*n* = 4), and enzyme‐linked immunosorbent assay (ELISA) (*n* = 4) 3 days after laparotomy. In the prevention study, mice received intraperitoneal injections of tauroursodeoxycholic acid (TUDCA, 200 mg/kg), an ER stress inhibitor, 30 min before laparotomy.^19^ The mice were randomly assigned to the Ctrl+Vehicle, Ctrl+TUDCA, A/S + Vehicle, or A/S + TUDCA groups. The hippocampus were harvested for western blotting (*n* = 4), immunofluorescence staining(*n* = 4), ELISA (*n* = 4) 3 days after laparotomy. The aged mice were used for neurocognitive function assessment starting day 4 after laparotomy (*n* = 9).

### 2.2 Establishment of mouse laparotomy model

The PND model was established by exploratory laparotomy under 1.4% isoflurane anesthesia, as previously reported.^20^, ^21^ Briefly, mice were anesthetized by inhalation of 2.0% isoflurane (100% oxygen flow, 1 L/min). After the mouse righting reflex disappeared, 1.4% isoflurane anesthesia was maintained (100% oxygen flow, 1 L/min).^22^ After skin preparation and disinfection, a stratified incision was made along the median abdominal line to create a 2.5‐cm longitudinal incision. The left and right rectus ophthalmic forceps were separated, and a mesangial arterial loop (3–5 cm) was gently pulled out of the ileum. The ileum (3–5 cm) was placed on the surface of a normal saline wet sterile gauze, and the exposed intestinal segment was covered to protect the incision. After the intestinal contents were removed, the exposed intestinal segment was evenly and gently twisted with the thumb, index finger, and middle finger for approximately 10 min and separated by gauze. The intestinal segment was gently kneaded to avoid mesenteric arteriovenous bleeding and severe mechanical tissue damage. The small intestine was reinserted, and the incision was closed using 4–0 absorbable sutures. Finally, a 5% lidocaine cream was used for analgesia after surgery. The procedure was conducted under sterile conditions and lasted approximately 30 min on a thermal blanket.

### 2.3 RNA‐seq

After RNA extraction and library construction, the Illumina NovaSeq 6000 and Fastp were used for RNA‐seq and assessment of the quality of the sequencing data; clean reads were consistent with the mouse genome (GRCm38/mm10) using Hisat2. The number of reads for each gene was counted by RSEM, and the FPKM of each gene was calculated. Differentially expressed genes (DEGs) were determined using DESeq2 and defined by a *p*‐value <0.05. Multi‐dimensional scaling (MDS) analysis was used to conduct dimensionality reduction analysis based on the expression of DEGs.

### 2.4 DEGs functional enrichment analysis

Enrichment analysis of DEGs following A/S was conducted using the web‐based platform Metascape (http://metascape.org). Metascape serves as an online bioinformatics pipeline designed for the analysis of multiple gene lists, facilitating comprehensive gene function annotation and decision‐making in data‐driven prioritization.^23^ Gene Ontology (GO) biological processes enriched by DEGs were examined to gain insights into the underlying biological changes associated with A/S.^24^

### 2.5 Hub gene screening

To investigate the genes closely associated with changes in biological processes in the hippocampus following A/S, we adopted an integrated approach using two hub gene screening methods. First, hub genes were selected from the protein–protein interaction (PPI) network of DEGs established using the STRING database by using 12 different algorithms within the CytoHubba application.^25^, ^26^ Second, we identified hub genes using weighted gene coexpression network analysis to detect highly correlated gene modules and hub genes within these modules.^27^ The soft threshold parameter (β) was set to 9, signifying strong correlations between genes based on a threshold value of 0.8, to construct a scale‐free network; the dynamic tree‐cutting algorithm was utilized to identify gene modules, resulting in 20 distinct modules following the merger of highly correlated coefficients; correlations between sample group phenotypes and modules were calculated, considering correlation coefficients >0.6 and *p*‐values <0.05; and hub genes within the selected modules were screened based on criteria such as an absolute gene significance value >0.8, module membership >0.8, and *q*‐weighted value <0.05. The intersection between PPI and module hub genes was recognized as a set of hub genes.

### 2.6 Quantitative reverse transcription (qRT)‐PCR

Total RNA was extracted using the Fast RNA Extraction Kit for Animals (RK30120; ABclonal, China) and reverse transcribed into cDNA using ABScript III RT Master Mix for qPCR with gDNA Remover (RK20429; ABclonal). PCR was performed using 2X Universal SYBR Green Fast qPCR Mix (RK21203; ABclonal). *GAPDH* was used as an internal control. The relative expression levels of the target genes were evaluated via the 2^−ΔΔCt^ method. The primer sequences are shown in Table 1.

**TABLE 1:** Primers used for mRNA quantification in this study.

Gene	Sequences of primer (5’ to 3’)
*Hspa5*	Forward AGGAGGAGGACA AGAAGGAGGATG Reverse TGAACACACCGACGCAGGAATAG
*Xbp1*	Forward GCAGCAAGTGGTGGATTTGGAAG Reverse CAAGCGTGTTCTTAACTCC TGGTTC
*GAPDH*	Forward AGGTCGGTGTG AACGGATTTG Reverse GGGTC GTTGATGGCAACA

### 2.7 Western blotting

The mice were anesthetized, and the hippocampal tissues were dissected, homogenized, and lysed in RIPA buffer (PC101; Epizyme, China) containing 1 mM protease inhibitor (GRF101; Epizyme). Western blotting was performed according to standard procedures, including electrophoresis and membrane transfer; 5% nonfat milk was used to block the PVDF membrane for 2 h at room temperature, with primary antibody incubation at 4°C overnight followed by secondary antibody incubation for 1.5 h at room temperature. All antibodies used in this study are listed in Table 2. These proteins were detected by chemiluminescence and quantified using ImageJ software (National Institutes of Health, Bethesda, Maryland, USA).

**TABLE 2:** Antibodies used in this study.

Antibody	Manufacturer	Item number	Dilution rate	Source
Anti‐GRP78	Proteintech	11,587‐1‐AP	1:3000	Rabbit
Anti‐XBP1	Proteintech	24,868‐1‐AP	1:1000	Rabbit
Anti‐NLRP3	Cell Signaling Technology	15101S	1:1000	Rabbit
Anti‐caspase‐1	Cell Signaling Technology	24232S	1:1000	Rabbit
Anti‐GAPDH	Proteintech	60,004‐1‐Ig	1:50000	Mouse
Anti‐β‐Actin	Proteintech	66,009‐1‐Ig	1:10000	Mouse
Anti‐Iba‐1	Abcam	ab289874	1:200	Goat
Anti‐GFAP	Cell Signaling Technology	3670S	1:400	Mouse
Anti‐goat IgG‐FITC	Abcam	ab6881	1:800	Donkey
Anti‐mouse IgG‐FITC	Servicebio	GB22301	1:200	Goat

### 2.8 Immunofluorescence staining

Three days after A/S, the mice were anesthetized with isoflurane, and their bodies were perfused through the heart, first with a solution of phosphate buffered saline, and then with 4% paraformaldehyde. The brains were removed and kept in a 30% sucrose solution at 4°C after fixing in 4% paraformaldehyde for 48 h. The brains were cut coronally into 40‐μm‐thick sections from bregma −1.70 to −2.30 by a freezing microtome and mounted on glass slides. This sections of the brain were then prepared and exposed to primary antibodies against goat Iba‐1 and mouse GFAP overnight at 4°C. This was followed by incubation with donkey anti‐goat IgG‐FITC and goat anti‐mouse IgG‐FITC in dark for 1.5 h at room temperature. All antibodies used in this study are listed in Table 2. Before mounting, the slides were stained with DAPI to stain the nucleus for 10 min. Images of the sections were obtained using an Olympus confocal microscope (FV1000; Olympus, Japan). The quantification was performed as described previously. Briefly, six sequential coronal slices of the dorsal hippocampus(from anterior‐to‐posterior in coronal)of each mouse were randomly selected, and the microscopic fields were acquired in the hippocampal CA1 region. The number of pixels per image with intensity above a predetermined threshold level was considered as positive stained areas and quantified by Image J (National Institutes of Health, Bethesda, USA). The intensity on six slices was averaged for each animal and then normalized by that of the control group. Cell numbers were counted manually using the Cell Counter plugin. Area sizes were measured and the number of cells was expressed as number of positive cells per mm^2^. The average for each individual animal was calculated and used for statistical comparison between groups. All quantitative analyses were performed in a blinded fashion.

### 2.9 ELISA

We measured the proinflammatory cytokines in hippocampal tissue, using the IL‐18 and IL‐1β Mouse ELISA Kits (E‐EL‐M0730 and E‐EL‐M0037; Elabscience, China), according to the manufacturer's instructions.

### 2.10 Behavior experiments

Smart video tracking software (Panlab, Harvard Apparatus, USA) was used to record and analyze results from the mouse open field and Barnes maze experiments. Behavioral tests are administered daily from 8 a.m. to 17 p.m. There was enough time given as a “wash‐off” between behavior tests.

### 2.11 Open field

Each mouse was carefully placed in an open field arena made of plastic (40 × 40 × 40 cm) and allowed to explore for 8 min. All movements and traces were recorded using a camera. The time spent in the central zone and average speed (m/s) were calculated blindly to evaluate the anxiety level and motor ability of mice.

### 2.12 Barnes maze

The Barnes maze paradigm was used to evaluate the dependence of spatial learning and memory on the hippocampus, as previously described.^20^ Briefly, the mice underwent continuous training for 4 days, with three sessions on day 5 and four sessions on days 6–8. On day 9, the escape chamber was removed, and the mice were allowed to move freely for 2 min. All interactions were recorded on video and analyzed without bias. The time taken by the mice to enter the escape chamber within 2 min was measured to assess their spatial reference learning and memory capabilities. The data were analyzed using the Panlab smart video tracking software.

### 2.13 Fear conditioning test

The Ugo Basile fear conditioning system relies on the hippocampus and was used to test contextual fear conditioning in mice. The test was performed according to the protocol described in a previous study.^28^ On habituation day (day 10), the mice were allowed to freely explore the conditioning chamber (17 × 17 × 25 cm) for 10 min. On the training day (day 11), the mice were placed in the conditioning chamber and received five‐foot shocks at an intensity of 0.7 mA for 2 s, with a time interval of 35–60 s between shocks. The mice were returned to their cages, and were returned to the same room 24 h later (on day 12) for 5 min to evaluate memory retrieval. Freezing movements were recorded. To avoid odor interference, 75% ethanol was used to clean the apparatus after each animal testing. The freezing time of each mouse was analyzed in a blinded manner.

### 2.14 Statistical analysis

The data are presented as the mean ± standard deviation (SD). GraphPad Prism (v.10.0; GraphPad Software Inc., La Jolla, California, USA) was used to conduct the statistical tests. Shapiro–Wilk test was used to check whether the data were normally distributed. The sample size for each experiment was described in the figure legends. The data were subjected to Student's *t*‐test for two groups, two‐way analysis of variance (ANOVA) followed with post hoc Tukey test for four groups. Two‐way repeated measures ANOVA was used to compare the data of Barnes maze training sessions between groups and within one group, respectively. Statistical significance was determined by evaluating *p*‐values (two‐tailed) <0.05.

## 3 RESULTS

### 3.1 Altered metabolic processes in the hippocampus following A/S

**FIGURE 1:**
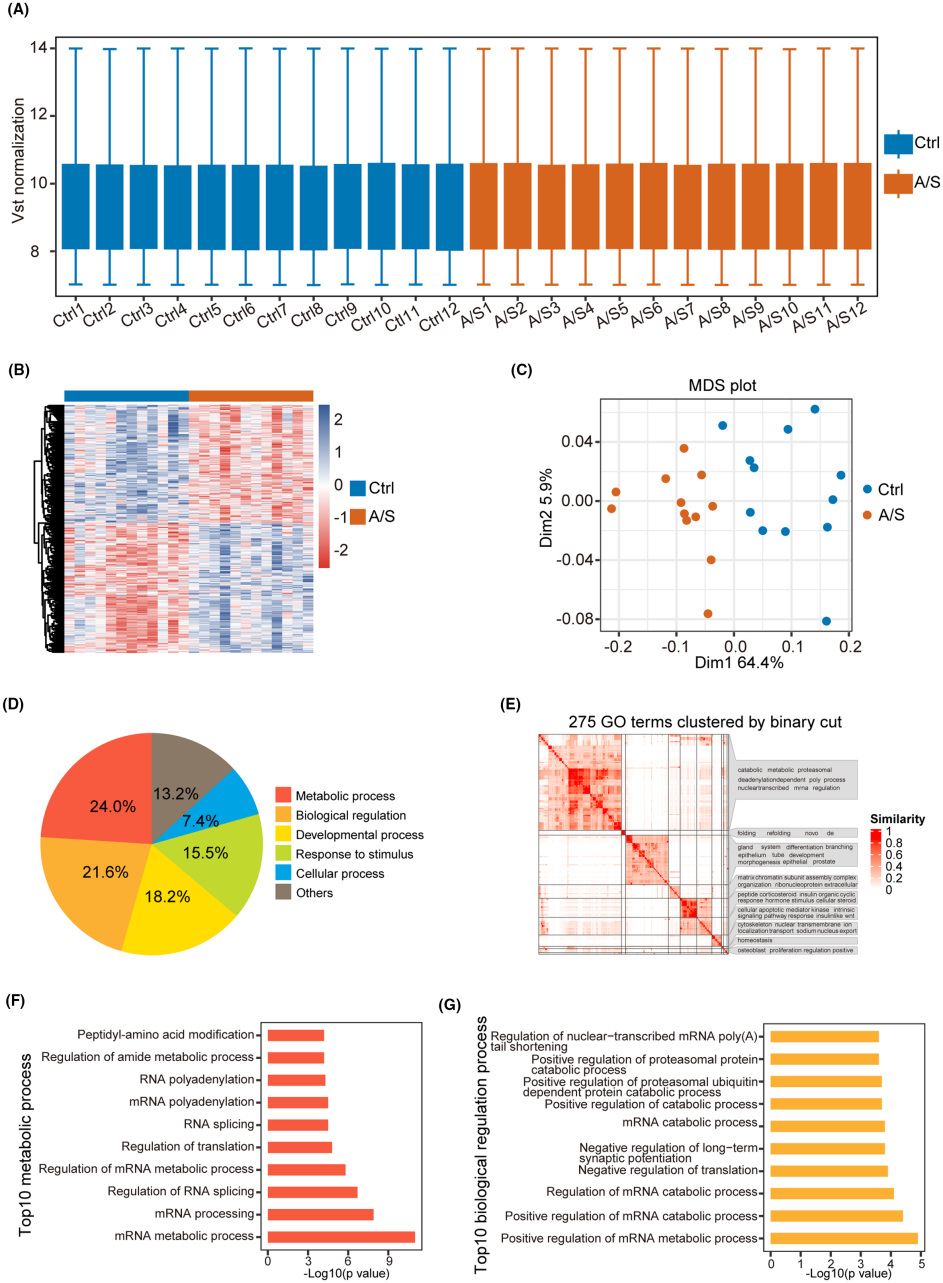
Function of DEGs associated with metabolic processes and biological process regulation in the hippocampus following A/S. (A) Boxplot of overall gene expression in each sample after normalization using the vst function. (B) Heatmap of DEGs between the A/S and Ctrl groups; 273 and 248 genes were upregulated and downregulated, respectively, in the perioperative neurocognitive disorder group. (C) DEGs divide all samples into two groups. (D, E) DEGs‐enriched biological process terms were associated with metabolic processes and biological regulation. (F) Top 10 biological processes related to the metabolic process. (G) Top 10 biological processes related to biological regulation.

Metabolic processes were altered in the hippocampus following A/S, as indicated by the GO enrichment analysis results. The overall gene expression for each sample is shown in Figure 1A. Compared with the Ctrl group, 273 and 248 genes were upregulated and downregulated in the A/S group, respectively. A heatmap displaying all the DEGs is shown in Figure 1B, and the DEGs can clearly distinguish between two groups (Figure 1C). GO enrichment analysis revealed that 24% and 21.6% of the enriched biological process terms were associated with metabolic processes and the regulation of biological processes, respectively (Figure 1D). The results also indicated that the enriched GO terms were associated with metabolic processes after clustering all GO terms (Figure 1E). The top 10 biological processes related to metabolic processes (Figure 1F) included those involved in mRNA metabolic processes, mRNA processing, and the regulation of RNA splicing. The top 10 biological processes related to the regulation of biological processes indicated that the DEGs were involved in regulating mRNA metabolic and catabolic processes (Figure 1G). Overall, the GO enrichment analysis results of DEGs suggested that metabolic processes and the regulation of biological processes were activated in the hippocampus following A/S in mice.

### 3.2 Hub genes associated with change after A/S

**FIGURE 2:**
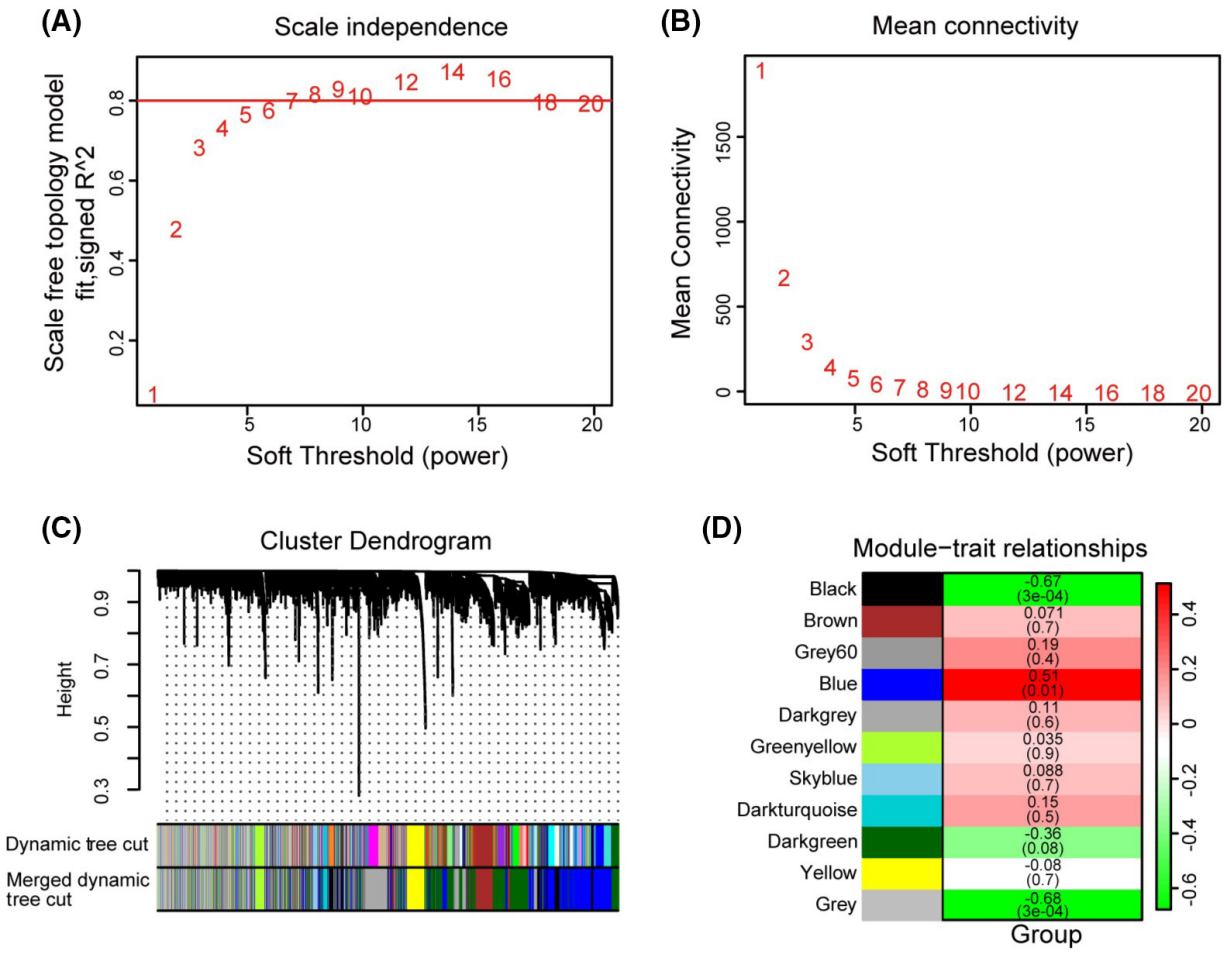
Weighted gene coexpression network analysis screened three modules significantly associated with A/S trait. (A, B) Scale independence and mean connectivity in all samples (*β* = 12). (C) Gene dendrogram and modules after merging in all samples. (D) Heatmap of the correlation between each module and A/S.

Four hub genes (*Hspa5*, *Igf1r*, *Sfpq*, and *Xbp1*) were identified using an integrated approach involving two methods. Initially, 23 hub genes in the DEGs were identified from the PPI network using the STRING database and CytoHubba program by using 12 different algorithms. The genes were screened using at least six algorithms (Table 3). For further analysis, transcriptomic data from 22,245 genes were used in conjunction with weighted gene coexpression network analysis. Samples were excluded based on standardized connectivity values <−5, and all 24 samples were included in the analysis. The optimal soft threshold parameter (β) was determined to be 12 for constructing the gene expression network (Figure 2A,B). Subsequently, 11 gene expression modules were obtained; modules with a correlation coefficient >0.75 were merged (Figure 2C).

**TABLE 3:** Times of top 30 hub genes selected from 12 algorithms with cytohubba in Cytoscape.

Genes	*n*	Genes	*n*	Genes	*n*	Genes	*n*	Genes	*n*
Hsp90b1	10	Pdgfrb	6	Abce1	3	Sox9	2	Ndufs2	1
Hspa5	10	Sfpq	6	Cep350	3	Srp54b	2	Pgam1	1
Creb1	9	Atp5a1	5	Creld2	3	Tlr7	2	Pkd2l1	1
Fus	9	Bms1	5	Dnajb4	3	Vtn	2	Pmp22	1
Hnrnpa2b1	9	Chd1	5	Hnrnpdl	3	Als2	1	Pnn	1
Hspa1b	9	Hnrnph1	5	Lars	3	Baz1b	1	Prpf38b	1
Notch1	9	Hnrnph3	5	Manf	3	Cd163	1	Rfxap	1
Ranbp2	9	Rps19	5	Med13	3	Cdc42ep1	1	Rnpep	1
App	8	Srsf10	5	Pdia6	3	Chst12	1	Sirt2	1
Cd4	8	Srsf5	5	Rbm14	3	Cnp	1	Slc25a12	1
Ep300	8	Srsf7	5	Zfp207	3	Creb5	1	Srp54a	1
Nipbl	8	Top1	5	2310022B05Rik	2	Ddx50	1	Tmem254b	1
Smg1	8	Dnaja1	4	Abl1	2	Dis3l2	1	Ubn2	1
Cdkn1a	7	Dnajc3	4	Ahsa2	2	Dmxl1	1	Upf3b	1
Xbp1	7	Fip1l1	4	Chordc1	2	Gpi1	1	Zc3h15	1
Xpo1	7	Pdia3	4	Dctn2	2	Il33	1	Zfp326	1
Ago2	6	Pdia4	4	Ilf2	2	Jak2	1	Zfp512b	1
Dnajb1	6	Ptges3	4	Mdn1	2	Mrc1	1	Zfp638	1
Hnrnpm	6	Sdf2l1	4	Prdm2	2	Mrps14	1		
Igf1r	6	Tra2a	4	Ranbp17	2	Mtrf1l	1		
Kmt2a	6	Ythdc1	4	Serpinb1a	2	Mycn	1		

**FIGURE 3:**
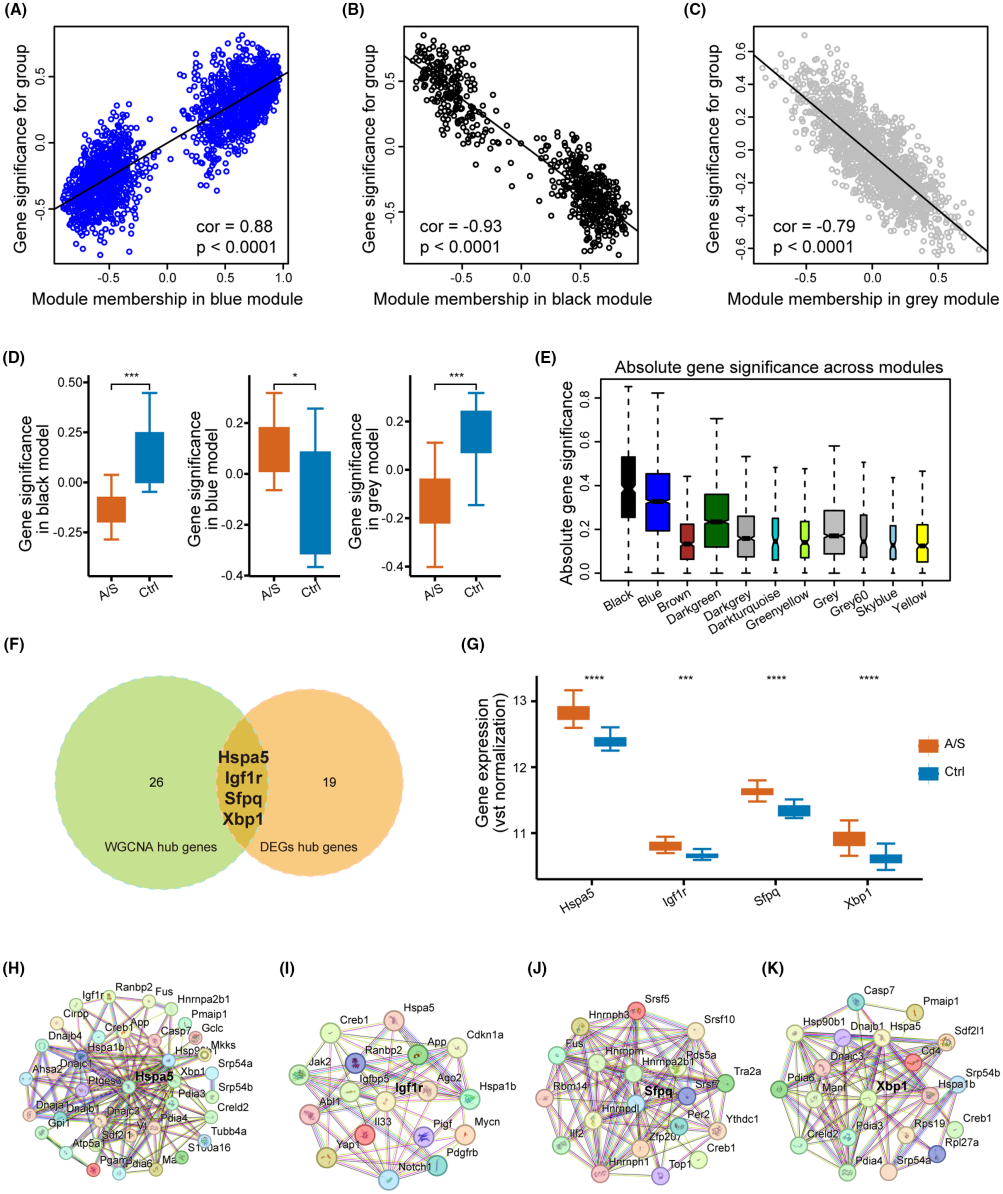
Four genes were screened as hub genes associated with the change after A/S. (A–C) Correlation between module membership and gene significance in three selected modules. (D) Difference of module eigengenes between the control group and A/S group in screened modules. (E) Boxplot of group trait‐based module significance (average gene significance in a module) across all modules. (F) Four hub genes were detected after merging two methods that were used to screen hub genes. (G) Boxplot of hub genes across the Ctrl and A/S group. (H–K) PPI network of the genes that interacted with four hub genes. Data are presented as the mean ± SEM with the presentation of data of each individual animal (*n* = 12). Results were analyzed by Mann–Whitney *U* test. ***p < 0.001, ****p < 0.0001.

To identify correlations between these modules and the experimental groups, we examined the absolute value of correlations >0.5 with a *p*‐value <0.05. Three modules closely correlated with the A/S group: blue (*R* ^2^ = 0.51, *p* = 0.01), black (*R* ^2^ = −0.67, *p* < 0.0001), and gray (*R* ^2^ = −0.68, *p* < 0.0001) were positively correlated (Figure 2D). Moreover, close correlations between gene significance and module membership were identified as blue (*R* ^2^ = 0.88, *p* < 0.0001), black (*R* ^2^ = −0.93, *p* < 0.0001), and gray (*R* ^2^ = −0.79, *p* < 0.0001; Figure 3A–C). Significant differences in gene expression between the Ctrl and A/S groups were observed in all three modules (Figure 3D). The absolute gene significance of the modules is shown in Figure 3E. Of 22,245 genes, 30 hub genes were finally identified based on stringent criteria (absolute gene significance >0.7, module membership >0.7, and q‐weight <0.01). Integrated with the two methods mentioned earlier, four hub genes (*Hspa5*, *Igf1r*, *Sfpq*, and *Xbp1*) were selected; all were upregulated in the A/S group (Figure 3F,G).

### 3.3 Elevated Hspa5 and Xbp1 gene expression and NLRP3 inflammasome activation after A/S

**FIGURE 4:**
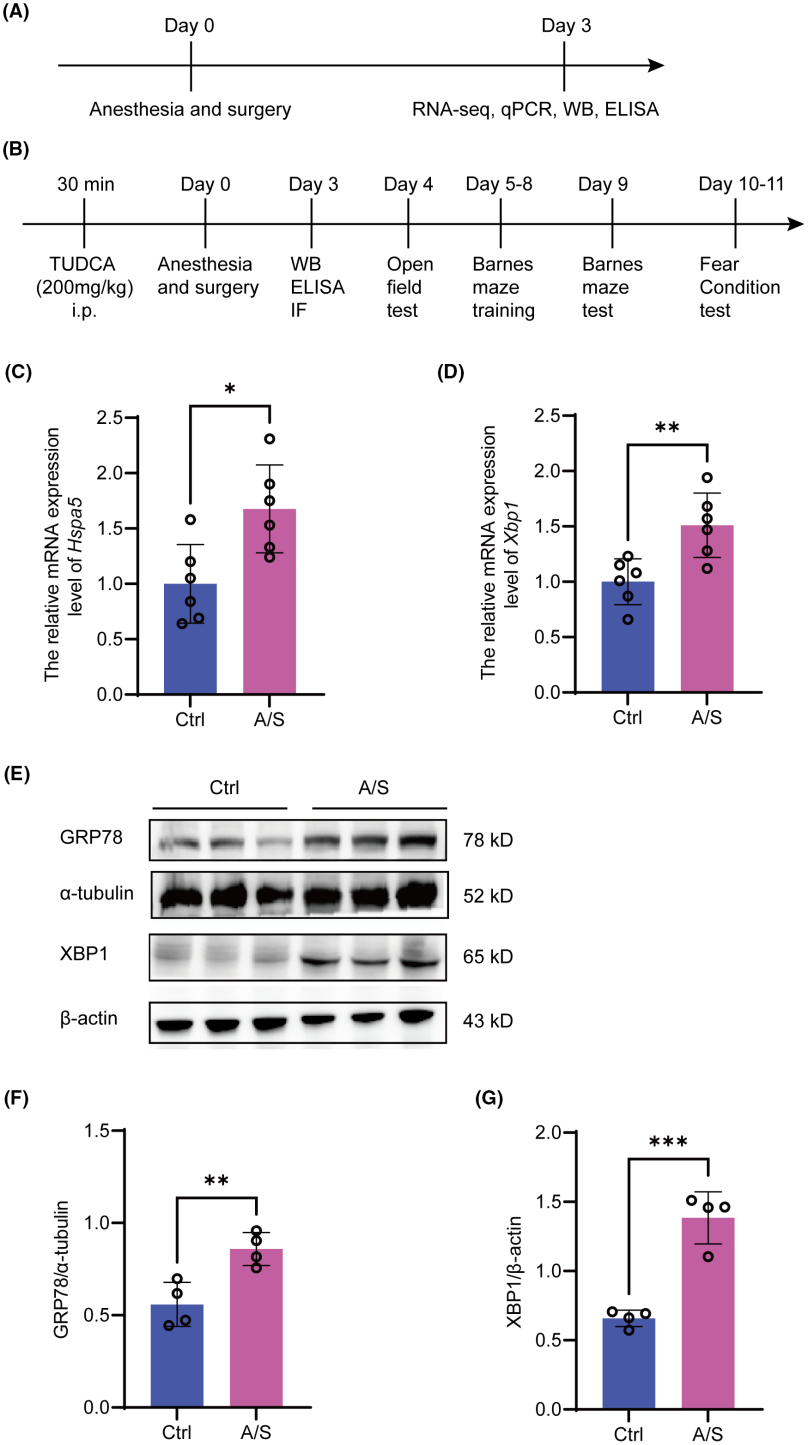
mRNA and protein expression level changes in the hippocampus of mice between the Ctrl and A/S groups. (A, B) Schematic diagram of the experimental procedure. A/S was performed on day 0. The mice were intraperitoneally injected with TUDCA (an ER stress inhibitor, 200 mg/kg) before exposure to isoflurane. Hippocampal tissue extraction, RNA sequencing, qPCR, WB, IF, and ELISA were performed on day 3. (C, D) mRNA expression levels of the *Hspa5* and *Xbp1* genes. (E) Representative western blots of GRP78 and XBP1 in the hippocampus; α‐tubulin and β‐Actin were used as controls, respectively. (F, G) Quantitative analysis of GRP78 and XBP1 expression. Data are presented as the mean ± SEM with the presentation of data of each individual animal (*n* = 6 for qPCR and *n* = 4 for western blot). Results were analyzed by *t*‐test. *p < 0.05, **p < 0.01, ***p < 0.001.

Following the RNA‐seq and bioinformatics analyses, the mRNA expression levels of *Hspa5* and *Xbp1* in the mouse hippocampus were remarkably upregulated after A/S. To verify this result, *Hspa5* and *Xbp1* mRNA and protein levels in the mouse hippocampal tissues of the two groups were detected by qPCR and western blotting. As shown in Figure 4C,D, compared with controls, the mRNA expression of *Hspa5* and *Xbp1* in the hippocampus significantly increased in the A/S group. Similarly, the protein expression levels of GRP78 (encoded by *Hspa5* gene) and XBP1, which play crucial roles in ER stress, showed the same trend (Figure 4E–G).

**FIGURE 5:**
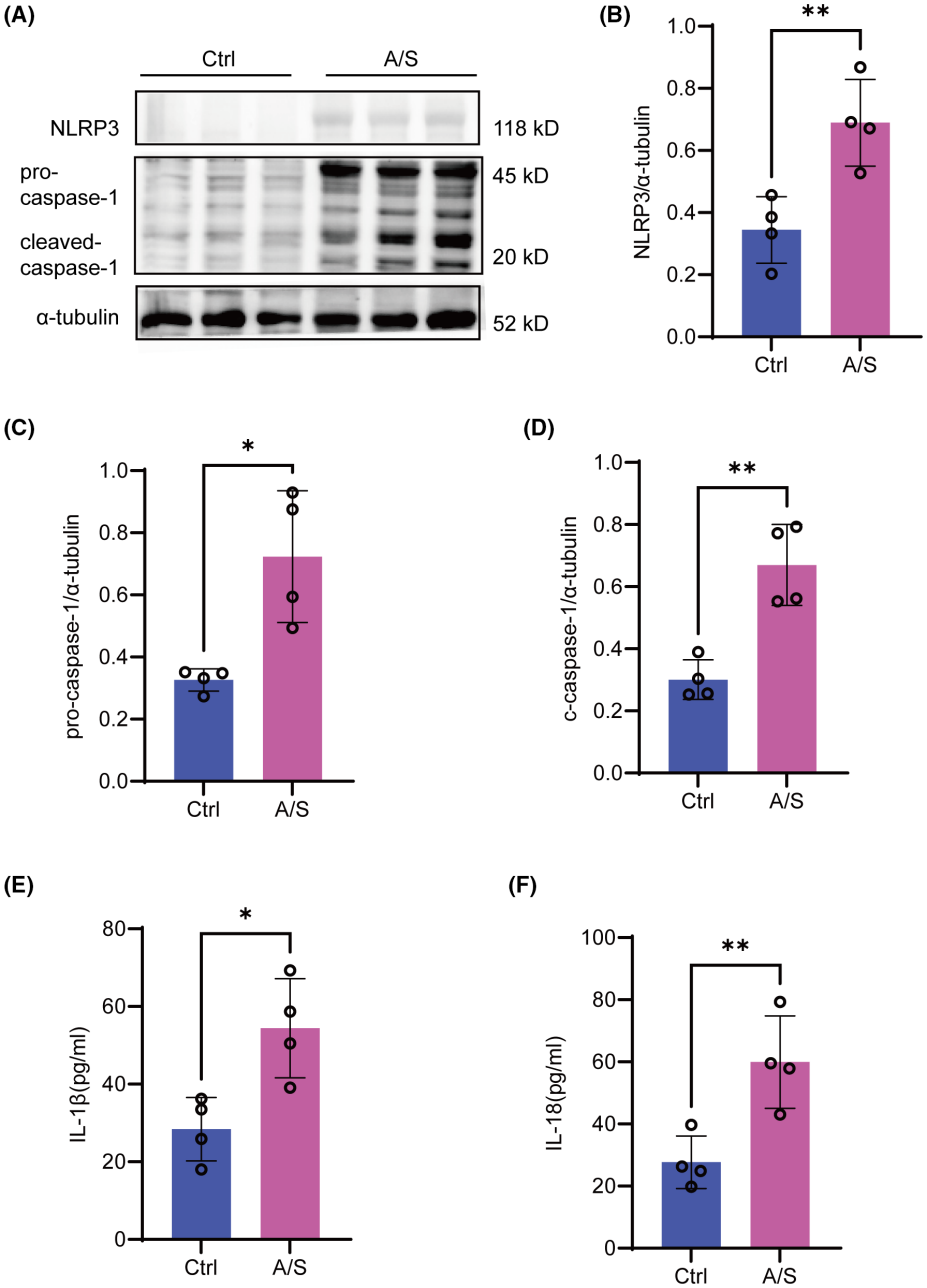
NLRP3 inflammasome in the hippocampus were obviously activated after anesthesia and surgery. (A) Representative western blots of NLRP3, pro‐caspase‐1 and cleaved‐caspase‐1 in the hippocampus; α‐tubulin was used as control. (B–D) Quantitative analysis of NLRP3, pro‐caspase‐1 and cleaved‐caspase‐1 protein expression levels. (E, F) Levels of inflammatory factors (IL‐1β, IL‐18) in the hippocampus. Data are presented as the mean ± SEM with the presentation of data of each individual animal (*n* = 4). Results were analyzed by t‐test. N.S.: No significant difference, *p < 0.05, **p < 0.01.

NLRP3 inflammasome activation is thought to play a vital role in neuroinflammatory processes.^29^ To observe NLRP3 inflammasome activation, NLRP3, pro‐caspase‐1, cleaved‐caspase‐1 expression, IL‐1β, and IL‐18 levels were detected by western blotting and ELISA; all significantly increased after A/S (Figure 5A–F).

### 3.4 ER stress inhibition attenuated NLRP3 inflammasome activation and neuroinflammation after A/S

**FIGURE 6:**
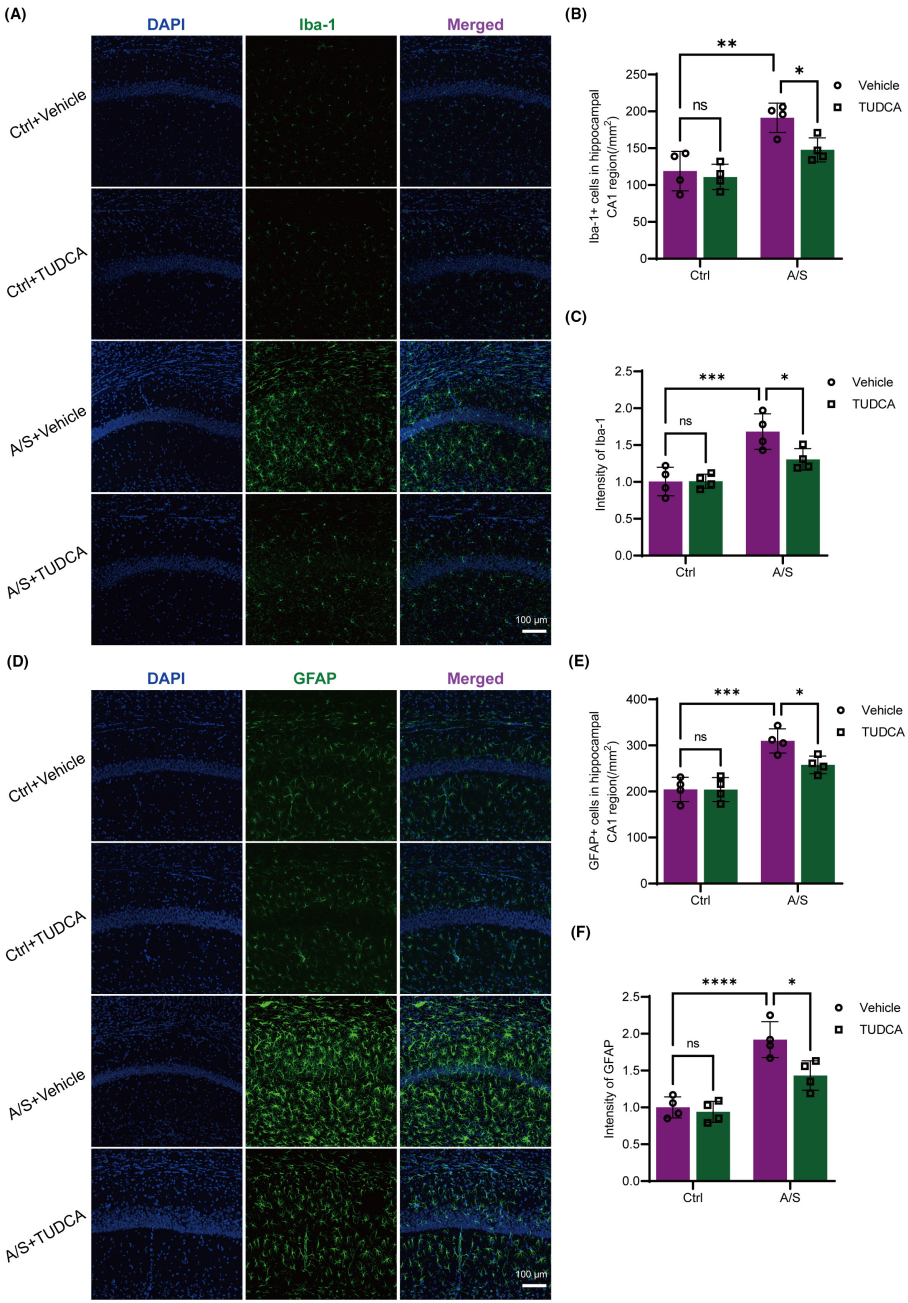
Activation of hippocampal microglia and astrocytes after A/S was detected and reversed by TUDCA preoperative administration. (A, D) Immunofluorescence staining images of Iba1 (A) and GFAP (D) in the hippocampal CA1 region of aged mice in four groups. Scale bar: 100 μm. (B, E) Comparison of the number of Iba‐1+ cells and GFAP+ cells in hippocampus of four groups of aged mice. (C, F) Comparison of fluorescence intensity of Iba‐1 and GFAP in hippocampal CA1 region of four groups of aged mice. Data are presented as the mean ± SEM with the presentation of data of each individual animal (*n* = 4). Results were analyzed by two‐way ANOVA followed with Tukey test. N.S.: No significant difference, *p < 0.05, **p < 0.01, ***p < 0.001, ****p < 0.0001.

**FIGURE 7:**
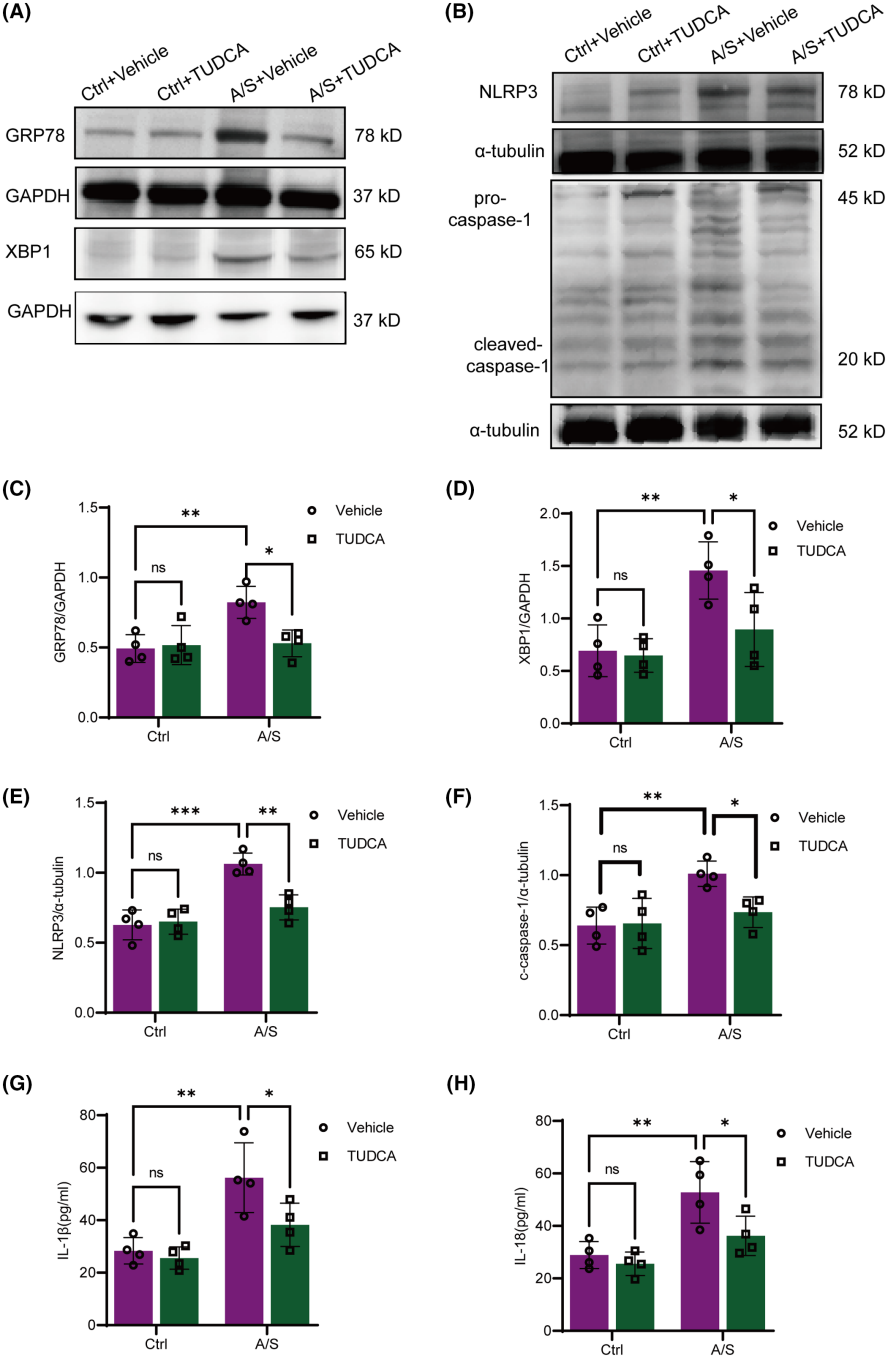
TUDCA preoperative administration inhibited NLRP3 inflammasome activation and neuroinflammation after anesthesia and surgery. (A, B) Representative western blots of GRP78, XBP1, NLRP3, pro‐caspase‐1 and cleaved‐caspase‐1 in the hippocampus. GAPDH and α‐tubulin were used as controls, respectively. (C–F) Quantitative analyses of GRP78, XBP1, NLRP3, and cleaved‐caspase‐1 protein expression levels. (G, H) Levels of inflammatory factors (IL‐1β, IL‐18) in mice hippocampus. Data are presented as mean ± SEM with the presentation of data of each individual animal (*n* = 4). Results were analyzed by two‐way ANOVA followed with Tukey test. N.S.: No significant difference, *p < 0.05, **p < 0.01, ***p < 0.001.

To clarify the role of ER stress in NLRP3 inflammasome activation and neuroinflammation after A/S, TUDCA was intraperitoneally injected into mice 30 min prior to laparotomy. The hippocampal microglia and astrocytes of aged mice in the A/S + Vehicle group were significantly activated, as shown in Figure 6, the number of Iba‐1 and GFAP positive cells and fluorescence intensity were significantly increased, which was reversed by TUDCA treatment (Figure 6A–F). Similarly, the expression of GRP78 and XBP1 in the hippocampus of aged mice significantly increased after A/S, and TUDCA prevention effectively inhibited the upregulation of these proteins (Figure 7A,C,D).

NLRP3 inflammasome activation also tended to be reversed in the A/S + TUDCA group. Specifically, the upregulation of NLRP3, cleaved‐caspase‐1 protein expression, and the inflammatory cytokines IL‐18 and IL‐1β levels after A/S were partially reversed after TUDCA prevention (Figure 7B,E–H).

### 3.5 ER stress inhibition ameliorated cognitive deficits after A/S

**FIGURE 8:**
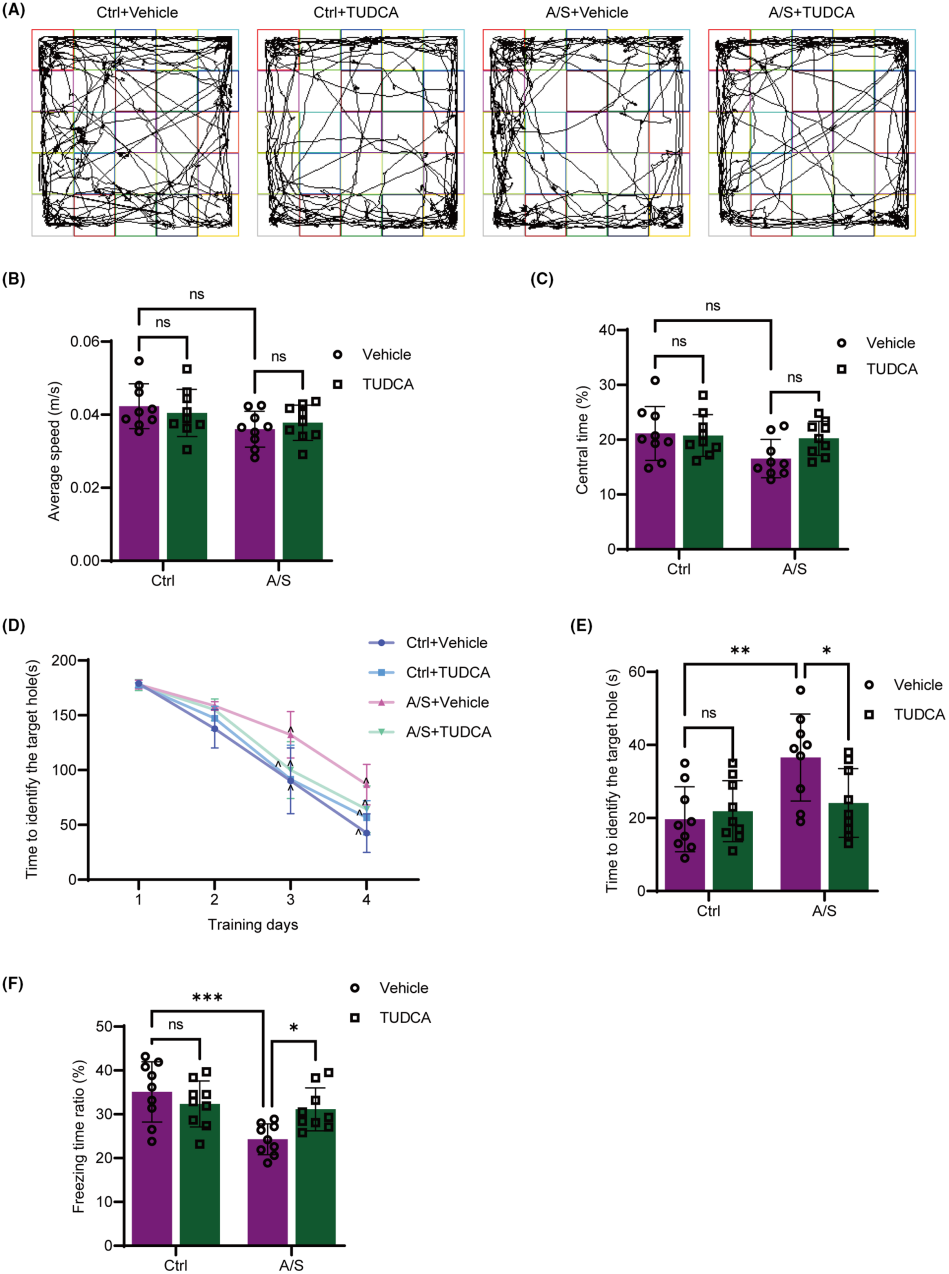
TUDCA preoperative administration attenuated postoperative cognitive decline in aged mice. (A–C) Representative tracks (A), average speed (B), and time spent in the central zone (C) of mice in the open field test. (D) Mean latency to identify the target hole of mice in the training phase of the Barnes maze tests (4 consecutive days). (E) Time to identify the target hole in the probe phase of the Barnes maze tests. (F) Ratio of freezing time of mice in the contextual fear conditioning tests. The data are expressed as the mean ± SD with the presentation of data of each individual animal (*n* = 9). Results were analyzed two‐way repeated measures ANOVA (panel D) and two‐way ANOVA followed with Tukey test (all other panels). N.S.: No significant difference, *p < 0.05, **p < 0.01, ***p < 0.001. ^*p* < 0.05 compared with the corresponding data on training day 1.

The open field test was used to assess motor activity and anxiety levels in mice. There was no significant difference in the average speed among the four groups (Figure 8A,B). Compared with the Ctrl+Vehicle group, the time spent in the central zone decreased significantly in the A/S + Vehicle group; however, there was no statistical difference between the other groups (Figure 8C).

Barnes maze and contextual fear conditioning tests were performed to evaluate the learning and memory abilities of mice. Mice in the A/S + Vehicle group displayed a longer latency to identify the target hole in the training and test phases of the Barnes maze compared with controls (Figure 8D,E) and exhibited a decreased freezing time in the fear conditioning test (Figure 8F) that was partially reversed by TUDCA prevention (Figure 8D–F).

## 4 DISCUSSION

In this study, we performed RNA‐seq analysis of the mouse hippocampus and screened four hub genes considered to play critical roles in neurocognitive disorders after A/S. Among these hub genes, *Hspa5* and *Xbp1* were related to ER stress and significantly upregulated. We observed learning and memory deficits in mice following A/S, accompanied by NLRP3 inflammasome activation and elevated levels of inflammatory cytokines. Preoperative administration of TUDCA downregulated the expression of GRP78 and XBP1 and inhibited NLRP3 inflammasome activation and neuroinflammation, thereby exhibiting an obvious therapeutic effect against postoperative cognitive decline.

Mediated by microglia and astrocytes, neuroinflammation is thought to be a key contributor to cognitive deficits.^7^, ^30^, ^31^, ^32^ Recently, the role of astrocytes in central nervous system (CNS) inflammation has received increasing attention.^33^, ^34^ Comprising the largest number of glial cells in the CNS, astrocytes maintain CNS homeostasis by interacting with neurons and other glial cells.^33^, ^35^ The metabolic cross‐dialogue between astrocytes and neurons interferes with inflammation, thereby enhancing neurotoxicity.^31^, ^36^ Specifically, lactate produced by glycolysis in astrocytes can be transported to neurons through the astrocyte‐neuron lactate shuttle to regulate cellular metabolism and neuroinflammation.^36^ Additionally, IL‐33 secreted by astrocytes promotes microglia‐dependent developmental synaptic pruning, highlighting the importance of astroglia‐microglia cross‐dialogue in the development of neural circuits.^31^ Bezzi et al.^37^ found that tumor necrosis factor (TNF)‐α secreted by microglia induces SDF‐1‐CXCR4 signaling to drive astrocytes to release glutamate and promote neuronal death. Similarly, TNF‐α, IL‐1α, and C1q secreted by microglia were shown to induce neurotoxic phenotypes in astrocytes.^38^

The NLRP3 inflammasome is closely associated with inflammatory responses.^39^ Accumulating evidence indicates that its activation is involved in various inflammation‐related diseases, including AD.^40^, ^41^ Cho et al.^12^ reported that dexmedetomidine, a sedative widely used in clinical practice, could alleviate postoperative cognitive deficits by inhibiting neuroinflammation mediated by the NLRP3 inflammasome. Similarly, Zhou et al.^42^ found that sevoflurane‐induced cognitive deficits in aged mice were mediated by NLRP3 inflammasome activation. Additionally, TL1A can promote A1 differentiation of reactive astrocytes via NLRP3 inflammasome activation, while aggravating postoperative cognitive decline.^43^ These results agree with our findings that cognitive dysfunction is associated with NLRP3 inflammasome activation. However, the trigger for such activation was unknown in these studies.

ER stress is a beneficial physiological response that serves to maintain protein homeostasis by activating the UPR.^44^, ^45^ However, an excessive ER stress response may damage the cells and tissues. This phenomenon has also been reported in patients with postoperative cognitive dysfunction.^46^ Sevoflurane A/S was demonstrated to induce ER stress, leading to learning and memory dysfunction in neonatal rats by causing autophagy dysfunction.^47^ Previous studies have indicated that ER stress in the hippocampus of mice is associated with the induction of postoperative cognitive decline.^48^ However, no additional information regarding the related molecular mechanisms is available.

ER stress regulates inflammatory signaling in various cell types, including macrophages, hepatocytes, and renal tubular epithelial cells.^49^ ER stress and inflammation are defensive and protective responses triggered by noxious stimuli; however, when intense or sustained, they can become destructive by regulating inflammatory mediators and triggering downstream pathways.^17^, ^50^ ER stress is a key factor involved in NLRP3 inflammasome activation.^17^, ^51^ As previously reported, FXR inhibits ER stress‐induced activation of the NLRP3 inflammasome in liver cells and alleviates liver injury.^52^ However, this has not been reported in nervous system disorders. The signaling pathway that acts as an initial trigger remains unclear; however, crosstalk between ER stress and NLRP3 inflammasome activation could occur, leading to postoperative cognitive dysfunction. To clarify this relationship, TUDCA an ER stress inhibitor, was administered to the mice prior to A/S. These results indicate that inhibiting ER stress can significantly reduce NLRP3 inflammasome activation‐mediated neuroinflammation and effectively alleviate postoperative cognitive deficits in aged mice.

Our study has some limitations; first, the molecular mechanism underlying ER stress‐induced NLRP3 activation was not elucidated. Moreover, the specific hippocampal cell types involved, including neurons, microglia, and astrocytes, wherein ER stress‐induced NLRP3 inflammasome activation occurred were not clear in this study. Additionally, inhibition of NLRP3 inflammasome activation and detection of ER stress‐related indicators should be performed to clarify the causal relationship between the two. Finally, neither NLRP3 inhibition nor knockout mice were used in this study. Further studies with detailed molecular mechanisms are required to validate our conclusions.

PND may be closely related to mitochondrial dysfunction, including excessive mitochondrial fission and mitophagy disorders. The ER and mitochondria are two organelles that are inseparable regarding structure and function.^53^, ^54^ The mitochondrial‐associated ER (MAM) is a dynamic membrane contact site between two important organelles that contain various proteins and play an essential role in many metabolic processes including lipid metabolism, calcium ion transport, mitochondrial fission, and autophagy.^53^, ^55^ Emerging evidence indicates that MAM is associated with various neurodegenerative diseases such as dementia, AD, and Parkinson's disease.^56^, ^57^, ^58^ Interestingly, coupling may occur between ER stress and mitophagy.^59^ Recent research demonstrates that platinum complexes inhibit tumor cells by inducing ER stress‐mediated mitophagy.^60^ Therefore, the crosstalk between ER stress and mitophagy in PND need further investigation.

In conclusion, our findings suggest that ER stress‐induced activation of the NLRP3 inflammasome mediates neuroinflammation and cognitive deficits following A/S, which can be effectively reversed by the administration of an ER stress inhibitor. These findings may provide a new perspective for researching the molecular mechanisms and elucidating the clinical prevention and treatment of PND, therefore benefitting older surgical patients.

## AUTHOR CONTRIBUTIONS

FM and JS contributed equally to this study. CL and HZ designed the study. FM and JS were responsible for establishing the animal model, conducting the molecular biology experiments, and writing the manuscript. FM, QJ, SC and ZX were responsible for analyzing the data, MZ conducted behavioral tests. HZ and XH performed bioinformatics analyses. CL and QL revised the manuscript accordingly. All the authors approved the final version of the manuscript.

## FUNDING INFORMATION

This work was supported by the National Natural Science Foundation [grant number: 82271223 and 82101256], Shanghai Municipal Committee of Science and Technology for the Program of Shanghai Academic/Technology Research Leader [grant number: 23XD1422900], Shanghai Municipal Health Commission [20244Z0007], Hongkou District Health Commission [HKLCFC202405, HKLCYQ202‐4‐04 and HKGWYQ202‐4‐07], Shanghai Fourth People's Hospital, School of Medicine, Tongji University [grant numbers: sykyqd01902, sykyqd06301, SY‐XKZT‐2022‐1006 and SY‐XKZT‐2022‐3004] and Medical Scientific Research Program of Hongkou District Health Committee [grant number: Hongwei2203‐09].

## CONFLICT OF INTEREST STATEMENT

The authors have no conflict of interest to declare.

## Supporting information


Data S1.

## Data Availability

The raw data generated from this study is available at https://www.ncbi.nlm.nih.gov/bioproject/1076499.
