# Supplementary figures and images for: Inhibiting endoplasmic reticulum stress alleviates perioperative neurocognitive disorders by reducing neuroinflammation mediated by NLRP3 inflammasome activation
Source: CNS Neurosci Ther. 2024 Oct 21;30(10):e70049. doi: 10.1111/cns.70049 (PMC11493103; doi:10.1111/cns.70049)

Full unedited gel/blot for Figure 4E, 5A

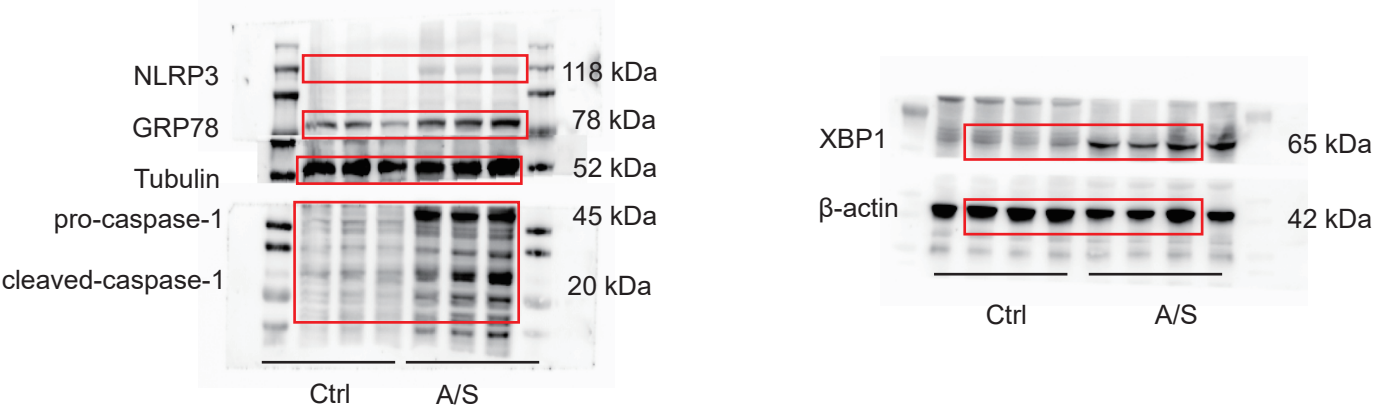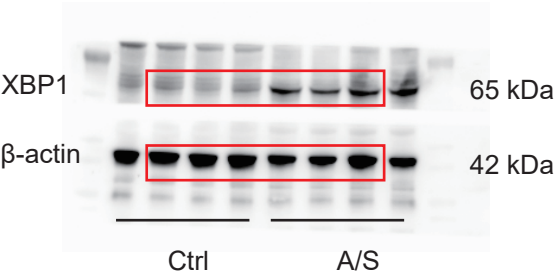

Full unedited gel/blot for Figure 7A, B

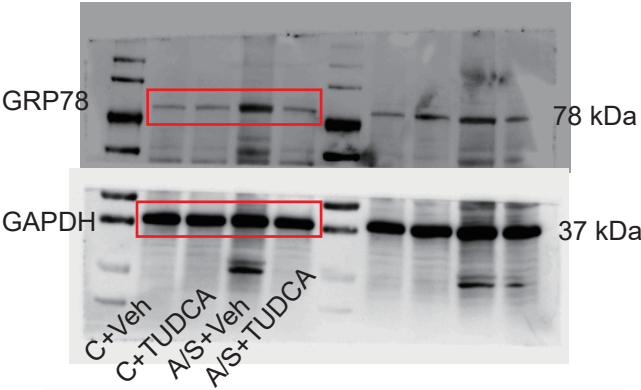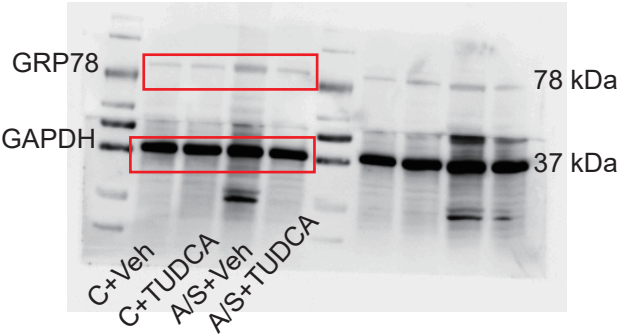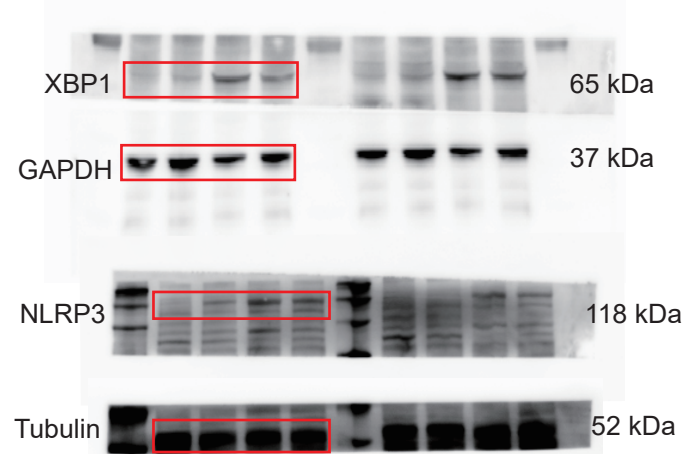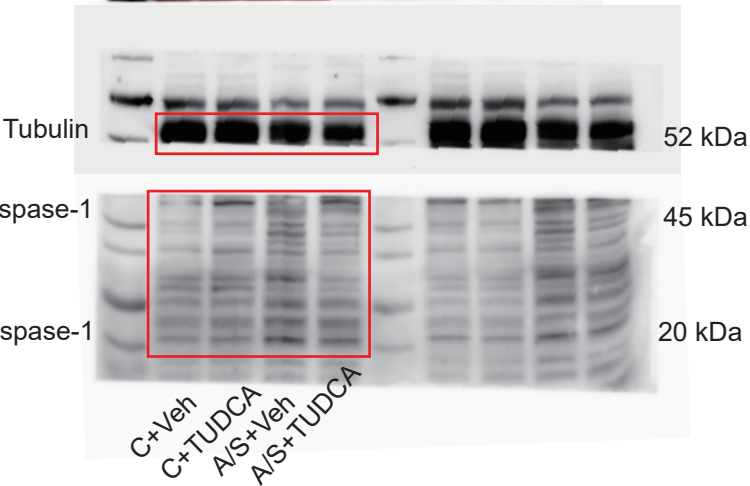

Supplement: Supplementary file 1 — Data S1. [file CNS-30-e70049-s001.pdf]
